# Targeting tumor suppressor p53 for organ fibrosis therapy

**DOI:** 10.1038/s41419-024-06702-w

**Published:** 2024-05-14

**Authors:** Yi-Ni Bao, Qiao Yang, Xin-Lei Shen, Wen-Kai Yu, Li Zhou, Qing-Ru Zhu, Qi-Yuan Shan, Zhi-Chao Wang, Gang Cao

**Affiliations:** School of Pharmacy, Zhejiang Chinese Medical University, No. 548 Binwen Road, Hangzhou, Zhejiang 310053 China

**Keywords:** Mechanisms of disease, Apoptosis, Chronic kidney disease

## Abstract

Fibrosis is a reparative and progressive process characterized by abnormal extracellular matrix deposition, contributing to organ dysfunction in chronic diseases. The tumor suppressor p53 (p53), known for its regulatory roles in cell proliferation, apoptosis, aging, and metabolism across diverse tissues, appears to play a pivotal role in aggravating biological processes such as epithelial-mesenchymal transition (EMT), cell apoptosis, and cell senescence. These processes are closely intertwined with the pathogenesis of fibrotic disease. In this review, we briefly introduce the background and specific mechanism of p53, investigate the pathogenesis of fibrosis, and further discuss p53’s relationship and role in fibrosis affecting the kidney, liver, lung, and heart. In summary, targeting p53 represents a promising and innovative therapeutic approach for the prevention and treatment of organ fibrosis.

## Facts

- p53 plays a crucial but complicated role in regulating organ fibrosis.
- p53-mediated cell senescence and apoptosis are required for organ fibrosis.
- The crosstalk between p53 and multiple cells regulates organ fibrosis.

## Open Question

- How p53 regulates the balance between cell proliferation and senescence in the course of organ fibrosis?
- Why the protein level and activity of p53 are differentially controlled in a cell type-specific manner during organ fibrosis?
- What are the potential problems and challenges for targeting p53 in treatment of organ fibrosis?

## Introduction

**Fig. 1:**
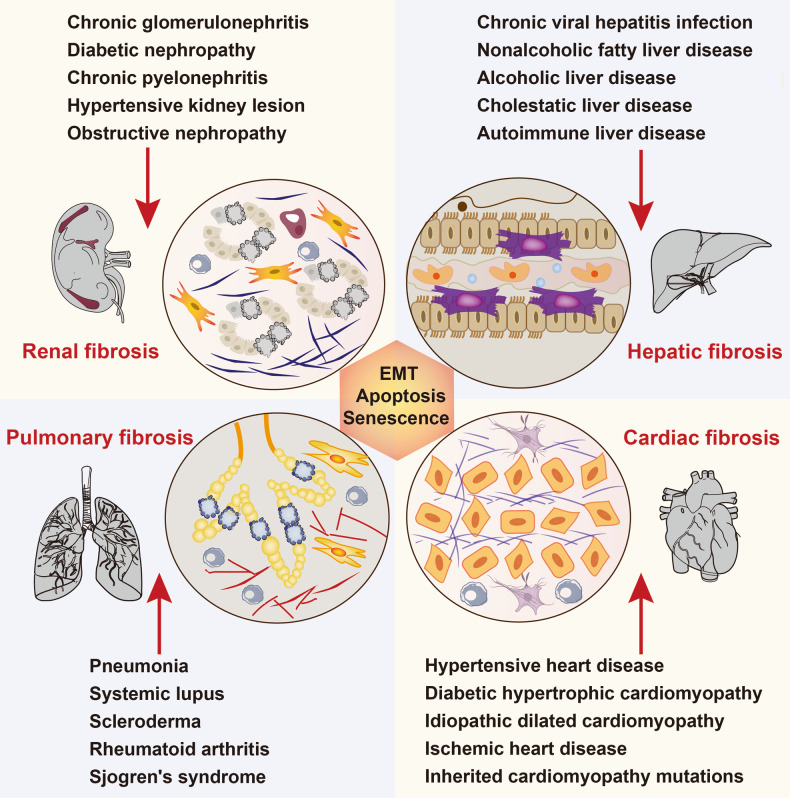
Schematic illustration of fibrosis pathogenesis in different organs. Noxious stimuli result in organ damage, inflammation, and fibrosis in the kidney, liver, lung, and heart. Fibrotic remodeling is implicated in numerous diseases and closely related to epithelial-mesenchymal transition (EMT), cell apoptosis, and cell senescence. Multiple functioning cells in different organs can be activated into myofibroblasts and further deposit large amounts of extracellular matrix, eventually leading to the formation of fibrosis.

Fibrosis is a pathological scarring process characterized primarily by chronic inflammation, excessive extracellular matrix (ECM) deposition, and activation of myofibroblasts [1]. Damage to tissues can result from various factors, including aging, inflammation, toxins, and infections. Following persistent tissue injury, the epithelium and endothelium trigger the release of inflammatory and immune mediators as part of the wound healing programs [2]. Activated fibroblasts and myofibroblasts are the crucial cellular effectors of fibrotic disease. Shortly after an initial inflammatory phase, quiescent resident fibroblasts transform into myofibroblasts, which significantly increase the production of ECM in damaged tissues [3]. When chronic injury and inflammation persist, ongoing wound-healing responses can evoke unrestrained tissue damage, repair, and regeneration, eventually leading to fibrosis in different organs such as kidney, liver, lung, and heart. (Fig. 1). Fibrotic remodeling is implicated in numerous diseases, including scleroderma, myocardial infarction, heart failure, cystic fibrosis, idiopathic pulmonary fibrosis (IPF), chronic kidney disease (CKD), diabetes, non-alcoholic steatohepatitis, and hepatitis, often resulting in organ dysfunction, failure, and even death [4]. Organ transplantation remain the only effective therapeutic option for end-stage diseases, unfortunately, donor organs are often in short supply. Despite extensive research on organ fibrosis, its pathogenesis has not been fully explained and the effective therapies are still lacking.

The tumor suppressor p53 (p53) is a member of sequence-specific nuclear transcription factors family, which includes two other members p73 and p63. Primitively, p53 is regarded as a type of tumor suppressor gene in the body that participates in the regulation of cell cycle arrest, apoptosis, senescence, ferroptosis, and autophagy to promote cell survival or limit cell malignant transformation [5]. It has been shown that p53 can modulate dynamin-related protein 1 mediated mitochondrial dynamics or directly activate the cellular senescence regulator p21 to accelerate cell cycle arrest and cell senescence [6, 7]. The B cell lymphoma-2 (Bcl-2) family proteins, including BAX, p53-upregulated modulator of apoptosis (PUMA), and NOXA, are key regulators for p53-dependent apoptosis checkpoints. Additionally, p21 and GADD45A have emerged as potent downstream mediators for p53-dependent cell cycle arrest [8]. The incidence of apoptosis is raised among most types of aging cells in the body [9]. Cell senescence and apoptosis have emerged as crucial predisposing factors for organ fibrosis [10, 11]. Numerous studies have demonstrated that p53 serves as master regulator of apoptosis and senescence, exerts regulating effects on fibroblast activation and ECM production, indicating that p53 plays a crucial role in the development of organs fibrosis.

In this review, we will focus on recent advances of p53 on organ fibrosis. Firstly, we will introduce the structure and function of p53, and then illustrate the underlying pathological mechanisms of organ fibrosis. In addition, we will discuss potential directions of p53-targeted therapy for fibrosis in different organs, which may provide potential ideas for the diagnosis and treatment of fibrotic diseases.

## p53

### Structure and function

The p53 gene contains multiple functional structural domains such as an N-terminal transactivation domain (TAD1 and TAD2, residues 1-40 and 40-60, respectively), a proline-rich domain (PRD, residues 60-90), a DNA-binding domain (DBD, residues 94-312), a C-terminal oligomerization domain (OD, residues 323-355), as well as a C-terminal domain (CTD, residues 364-393). Typically, the promoter response element DNA binding by p53 is an essential early step for transcriptional activation [12]. TAD1 and TAD2 involve several phosphorylation sites, which can modulate degradation and activity of p53 in response to stress stimulus [13]. In addition, the inactivation of TAD1 and TAD2 has been shown to play a role in directing p53 target gene selection, suppressing the growth of tumor, and promoting cellular senescence [14, 15]. Mutations or deletions in the PRD can regulate p53 degradation, transactivation function, and growth suppression [16, 17]. The following DBD is required for the definition of primary DNA-binding specificity. Besides, it can also carry numerous hot-spot mutation sites to control tumor suppressive transcription factor activity [18, 19]. Notably, CTD is highly unstructured and harbors primary sites for acetylation, methylation, and phosphorylation. These modifications are directly involved in p53 functions regulation in all aspects, such as transcriptional activity, protein stability regulation, co-factors recruitment, and complex DNA-binding behavior [20, 21].

### Activation mechanism

The p53 is activated in response to a range of stimuli, including DNA damage, hypoxia, oncogene activation, and ribosomal stress. The activation involves a three-step sequential process, which includes p53 stabilization, derepression, and promoter-specific activation [22]. Many mechanisms such as genetic (mutation, single-nucleotide polymorphism), transcriptional (epigenetic inhibition of p53 transcription), mRNA (alternative splicing), and protein (protein folding, localization)-level regulation, have been shown to regulate p53 activation and its function [5]. Notably, post-translational modifications (PTMs) can regulate p53 stability, conformation, localization, binding partners and are the most important mechanism to modulate p53 levels and activity [23]. There are more than 300 different PTMs of p53, mainly include acetylation, phosphorylation, methylation, and ubiquitination. It has been confirmed that TIP60 acetyltransferase mediates p53 acetylation at K120, which is required for p53-dependent cell growth arrest and apoptosis [24, 25]. Hepatocyte odd protein shuttling is a novel shuttling protein that facilitates p53-dependent mitochondrial apoptosis by inhibiting the proteasomal degradation of p53 [26]. The E3 ubiquitin ligase murine double minute 2 (MDM2) and its homolog murine double minute 4 (MDM4) are the two primary negative regulators of p53, which play essential roles in ferroptosis, DNA repair, and senescence regulation [27, 28]. MDM2 not only promotes ubiquitylation and proteasomal-dependent degradation of p53, but also binds to p53 to directly inhibit its transcriptional activity [29]. *N*‐acetyltransferase 10 promotes p53 activation by acetylating p53 at K120 and counteracting MDM2 action [30]. The phosphorylation of p53 on S15, T18, and S20 residues disrupts the binding of p53-MDM2, leading to aberrant p53 regulation and aging phenotypes [31, 32]. In addition, MDM4 has been shown to interact with MDM2 to reverse MDM2-mediated p53 degradation while maintain suppression of p53 transactivation [33].

## Pathogenesis of fibrosis

Fibrosis is a progressive medical condition and an outcome of severe tissue damage or wound healing disorders. The pathological mechanisms underlying fibrosis in various organs primarily encompass epithelial-mesenchymal transition (EMT), cell apoptosis, and cell senescence.

### EMT

There are several basic types of epithelial cells such as secretory, ciliated, club, goblet, and basal cells, which are crucial to maintain tissue homeostasis in various organs [34]. During fibrotic process, organs are exposed to chronic and continuous stimuli, epithelial cells can undergo a great diversity of changes in response to injury. Damaged cells dedifferentiate into mesenchymal states (EMT), which accompanied by the loss of apical-basal polarity and adhesion [35]. The EMT plays a fundamental role in the initiation of organ fibrosis. The progression of EMT is modulated by Snail, Twist, and zinc-finger E-box-binding (ZEB) transcription factors, these factors can restrain epithelial genes and activate mesenchymal phenotype markers [36]. Coincident with the downregulation of epithelial markers such as E-cadherin, occludin, claudin-1, the expression of mesenchymal markers like N-cadherin, fibronectin (FN), vimentin, α-smooth muscle actin (α-SMA) are significantly upregulated, all of which ultimately accelerate the transformation for epithelium into mesenchymal cells [37]. From a mechanistical perspective, EMT is orchestrated by multiple pathways, such as transforming growth factor beta (TGF-β), Notch, Hedgehog, and Hippo family proteins [38]. Notably, EMT can further promote the expression of profibrotic factors, including connective tissue growth factor (CTGF) and collagen I, ultimately leading to ECM deposition and organ fibrosis.

### Cell apoptosis

Cell apoptosis is a type of programmed cell death and is a key regulatory process as a defense mechanism to maintain internal stability in an organism [39]. There are currently three pathways for cell apoptosis, such as the death receptor apoptosis pathway, the mitochondrial apoptosis pathway, and the endoplasmic reticulum apoptosis pathway [40]. Cells undergo apoptosis exhibit a battery of characteristic changes including nuclear condensation, cell shrinkage, membrane blebbing, and DNA fragmentation [41]. A recent study has found that p53 is an essential regulator of cell apoptosis. As a nuclear transcription factor, p53 transcriptionally activates pro-apoptotic genes such as BAX, NOXA, and PUMA, and inhibits anti-apoptotic Bcl-2 family proteins [42]. In addition, p53 can migrate to the mitochondria, interact with Bcl-2 family proteins to accelerate mitochondrial outer membrane permeabilization and facilitate cytochrome c (Cyt c) release. Cyt c binds to apoptotic protease activating factor 1 and subsequently activates caspase cascade in the cytosol, thereby leading to mitochondrial apoptosis [43, 44]. In the fibrotic process, chronic impairment can lead to epithelial cells apoptosis, the fibroblasts have a major function in resisting apoptosis [45, 46]. Fas/FasL-mediated apoptosis of hepatocytes has been identified as an important factor that leads to liver fibrosis [47]. Therefore, targeting apoptosis of specific cell types such as epithelial cells and hepatocytes may provide a novel approach for the prevention and treatment of fibrosis.

### Cell senescence

Cellular senescence is a stress-inducible cellular state, which characterized by morphological and metabolic changes, chromatin reorganization, altered gene expression, and adoption of a pro-inflammatory phenotype known as senescence-associated secretory phenotype (SASP) [48]. There are many different stressful stimuli, ranging from oncogene activation, DNA damage to oxidative stress, all of which eventually can result in premature senescence. Telomere dysfunction caused by telomere shortening or altered telomere structures has been proposed as a crucial molecular feature for cell senescence [49]. Telomeres are composed of repetitive DNA sequences of TTAGGGn and a six-member protein shelterin complex. Telomere length is generally maintained by an enzyme called telomerase, and impaired telomerase activity can lead to telomere shortening. Telomere shortening initiates a persistent DNA damage response (DDR), which in turn drives senescence and chromosome instability. Therefore, persistent DDR activation is one of the root causes for cellular senescence [50]. Moreover, persistent DDR can promote the activation of p53 and p21, thereby leading to cell cycle arrest [51]. The p53 is a central regulator of DNA damage and can control DNA damage checkpoint via regulating cell cycle arrest and apoptosis [52, 53]. Cellular senescence has emerged as a critical contributor to numerous fibrotic diseases [54–56]. Various types of cells, including epithelial cells, endothelial cells, fibroblasts, and macrophages, have been shown to exhibit a senescence-like phenotype during tissue fibrosis. The senescent epithelial cells secrete numerous SASP factors including TGF-β, which then induce interstitial fibroblast proliferation and differentiation into myofibroblast. The differentiation of fibroblast can stimulate excessive ECM deposition and further promote organ fibrosis [57, 58].

## Roles of p53 in organ fibrosis

### Roles of p53 in renal fibrosis

Renal fibrosis is the final manifestation for the transformation of acute kidney injury to CKD. The global morbidity and mortality of CKD are growing rapidly in recent years, the current therapies for CKD are confined to dialysis or kidney transplantation, indicating that current pharmacological treatments have limited efficacy in clinic [59]. Renal tubular epithelial cells (RTECs) are the kidney-resident cell within tubulointerstitium and exert an actively role in renal fibrosis following injury. In fibrosis development and progression, activated fibroblasts or myofibroblasts are the dominating contributors to produce ECM in the kidney [60]. The myofibroblasts are derived from resident fibroblasts, pericytes, bone marrow-derived cells, especially polarized tubular epithelial cells in injured tissues [61]. A fibrogenic niche is a particular tissue microenvironment that spontaneously accelerates fibroblast activation in organ fibrosis. Polarized tubular epithelial cells and immune cells are the primary effector cell types within fibrotic niche of kidney [62, 63]. There is a rising prevalence of glomerulosclerosis and interstitial fibrosis in the aging kidney. Similarly, chronic accumulation of multiple senescent cells in renal diseases can also contribute to progressive renal fibrosis after injury [64, 65].

Unilateral ureteral ligation (UUO) and ischemia-reperfusion injury (IRI) are two common models of renal fibrosis. Notably, p53 protein level is distinctly upregulated in the fibrotic kidneys of UUO and IRI mice, which accompanied by increased α-SMA, CTGF expression, and substantial RTECs arrested at G2/M. The increased cell cycle arrest can accelerate SASP secretion and EMT program during fibrosis. Further research found that knockdown of p53 significantly attenuates UUO-induced renal fibrosis, while its overexpression aggravates fibrosis [66]. Importantly, p53 is highly expressed in epithelial cells of the developing kidney and significantly upregulated in proximal tubule epithelial cells of patients with CKD [67, 68]. Ying et al. found that proximal tubule cell-specific knockout of p53 can attenuate IRI and repeated low-dose cisplatin-induced renal fibrosis by inhibiting cell necrosis, apoptosis, and inflammation [69, 70]. In addition, diabetes has emerged as one of the risk factors for renal fibrosis. Both pharmacological inhibition and proximal tubule cell-specific knockout of p53 can alleviate tubular epithelial disruption, renal dysfunction, and fibrosis in streptozotocin-induced diabetic nephrology mice. On the one hand, p53 binds to lncRNA zinc-finger E-box binding homeobox1-antisense RNA 1 (ZEB1-AS1), significantly inhibits ZEB1-AS1 and ZEB1 expression to promote ECM accumulation. On the one hand, p53 can also directly bind to miR-214 for its transcription, and suppress unc-51–like autophagy-activating kinase 1 (ULK1) expression to trigger autophagy impairment. Both ECM accumulation and autophagy impairment can further aggravate renal fibrosis [71, 72]. Another study indicated that tumor necrosis factor α-induced protein 8 (TNFAIP8) capsulated in RTECs-derived exosomes can promote the degradation of p53 in fibroblasts. The decreased p53 not only suppress fibroblast apoptosis through inhibiting Bax and caspase 3, but also promote fibroblast activation and proliferation by triggering the de-repression of cyclin D1 and c-Myc, all of which contribute to renal fibrosis [73].

**Fig. 2:**
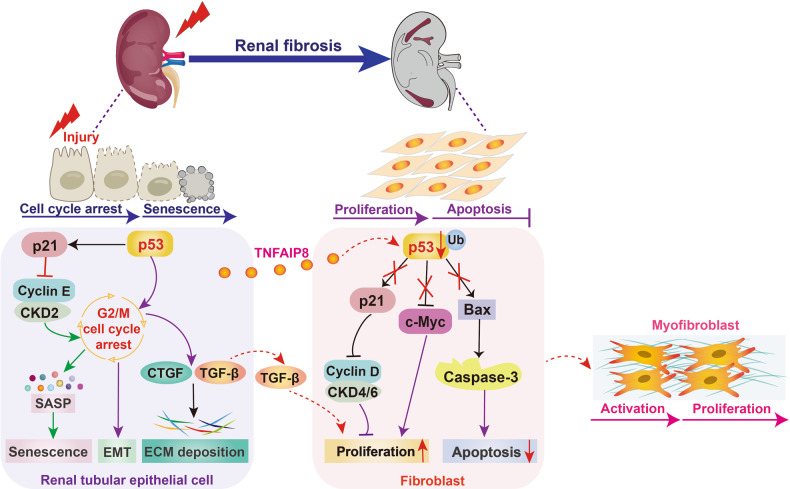
Roles of p53 in renal fibrosis. In response to nociceptive stimulus, p53 is activated in the renal tubular epithelial cells to accelerate cell senescence, whereas inactivated in renal fibroblasts delays cell apoptosis, all of which promote the activation of myofibroblasts and renal fibrosis.

Importantly, acetylation and phosphorylation-mediated p53’s PTMs also play an important role in renal fibrosis. TGF-β can facilitate the phosphorylation and acetylation of p53 to stimulate the p53-SMAD3 complexes assembly, ultimately leading to renal fibrosis [74, 75]. In calcium oxalate deposition-induced rats, p53 acetylation levels are significantly increased, which exacerbates renal fibrosis by activating SLC7A11/GPX4 inactivation-mediated ferroptosis [68]. Moreover, p53 is a crucial substrate of sirtuin1, which protects against DDR-induced renal tubular cell senescence by deacetylating p53. A recent study in vivo and in vitro has proved that N-acetylcysteine can promote sirtuin1 activation and p53 deacetylation to attenuate cisplatin-induced renal tubule cell senescence and fibrosis [76]. In conclusion, the above evidence suggests that p53 plays a key role in the pathogenesis of renal fibrosis (Fig. 2).

### Roles of p53 in hepatic fibrosis

Hepatic fibrosis is a dynamic process characterized by excessive and imbalanced deposition of ECM [77, 78]. Advanced hepatic fibrosis leads to cirrhosis and end-stage liver diseases, but liver transplantation remains the only available approach in clinics [79]. Hepatotoxic injury and cholestatic injury-induced chronic inflammation is the main driving factors during hepatic fibrosis [80]. Following liver injury, the injured hepatocytes trigger inflammatory reaction via activating resident liver macrophages. The activated macrophages secrete various cytokines, including reactive oxygen species and TGF-β, to accelerate the activation and differentiation of quiescent hepatic stellate cells (HSCs) into myofibroblasts, which in turn promote abnormal ECM deposition and hepatic fibrosis [81, 82]. Several studies have pointed out that the senescent hepatocytes produce more SASP factors to promote HSC activation and hepatic fibrosis [83]. While the senescent activated HSCs remain metabolically active, exert an anti-fibrosis effect by reducing ECM components biosynthesis [84].

**Fig. 3:**
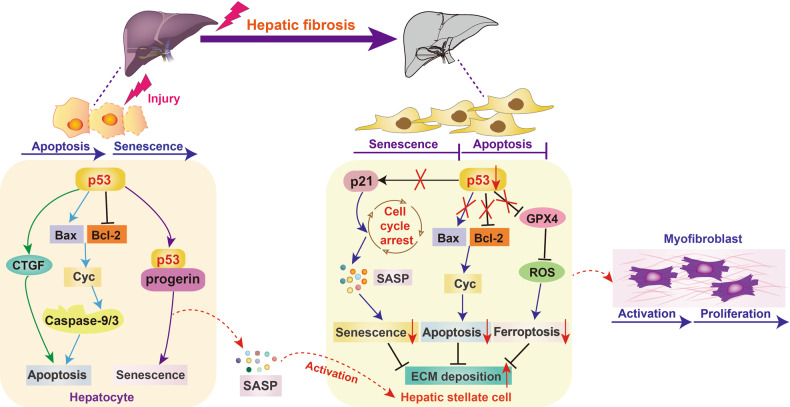
Roles of p53 in hepatic fibrosis. Activation of p53 in the hepatocytes triggers cell apoptosis, whereas p53 inactivation in hepatic stellate cells inhibits cell senescence and apoptosis through the regulation of related pathway, ultimately resulting in myofibroblast activation and hepatic fibrosis.

Numbers studies have revealed that elevated p53 in the hepatocytes is closely related to fibrotic liver diseases. Kodama et al. found that hepatocyte-specific MDM2 knockout mice showed a spontaneous liver fibrosis effect, which accompanied by endogenous p53 accumulation in liver tissue and hepatocytes. Nevertheless, the spontaneous liver fibrosis can be abolished in hepatocyte-specific MDM2/p53-double-knockout mice. Consistent with this, hepatocyte-specific knockout of p53 significantly inhibits CTGF synthesis and hepatocyte apoptosis, thereby alleviating atherogenic diet, thioacetamide injection, and hepatotoxin carbon tetrachloride (CCl4)-induced hepatic fibrosis [85, 86]. Insulin-like growth factor-1 (IGF-1) is a cytoprotective hormone that mainly synthesized in liver and also involves in cell senescence regulation. Luo et al. reported that IGF-1 therapy inhibits p53 nuclear translocation and nuclear p53-progerin interaction, which in turn relieves hepatocyte premature senescence and alleviates CCl4-induced hepatic fibrosis [87]. These results suggest that p53-dependent hepatocyte apoptosis and senescence play key roles in the occurrence and progression of hepatic fibrosis. Recently, emerging studies revealed that telomere dysfunction leads to sirtuin repression through p53-dependent mechanisms. Nicotinamide mononucleotide is a precursor of nicotinamide adenine dinucleotide and has been shown to stabilize telomeres. Treatment with nicotinamide mononucleotide significantly relieves telomere-dependent hepatic fibrosis in a partially sirtuin1-dependent manner [88]. The activation of HSCs is a central event during hepatic fibrosis. Ferroptosis is a new type of iron-dependent non-apoptotic cell death, inhibition of p53-mediated HSC ferroptosis has been shown to promote hepatic fibrosis by triggering ECM deposition and HSC activation [89–91]. Lujambio et al. found that in CCl4-treated mice, HSC-specific deletion of p53 exhibits an excessive accumulation of collagen production and an overall decreased senescent HSCs, indicating that p53 accumulation in HSCs can ameliorate hepatic fibrosis by promoting HSC senescence [92]. Another study also revealed that IGF-1 can accelerate HSC senescence in a p53-dependent manner to attenuate hepatic fibrosis [93]. In conclusion, these studies demonstrate that hepatic fibrosis formation in a p53-dependent manner within different cell types can lead to different outcomes (Fig. 3).

### Roles of p53 in pulmonary fibrosis

Pulmonary fibrosis is a chronic interstitial lung disease. The progressive and non-reversible destruction of lung architecture can result in lung scarring and severe shortness of breath [94]. IPF is the most frequent interstitial pulmonary fibrosis of obscure etiology, and there is no specific treatment for IPF. The hallmark of IPF is abnormal activation of alveolar epithelial cells (AECs), which lead to abundant accumulation of activated lung fibroblasts. The AECs are mainly composed of two cell types, alveolar epithelial type 1 (AT1) and alveolar epithelial type 2 (AT2). In particular, AT2 cells can differentiate into AT1 cells to restore normal alveolar architecture and function in response to continuous injury. The activated fibroblasts trigger alveolar-capillary basement membrane disruption, followed by excessive ECM production and a transformation toward profibrotic [95]. In addition, cellular senescence is a key pathological feature of IPF lung and senescence markers such as p21 and p16 are significantly upregulated along with disease severity. The senescent AECs promote pulmonary fibrosis by inducing alveolar epithelial dysfunctions, while senescent lung fibroblasts inhibit progressive fibrogenic reaction [96, 97].

**Fig. 4:**
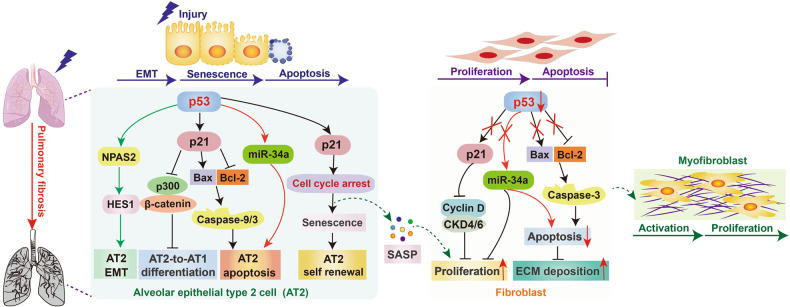
Roles of p53 in pulmonary fibrosis. Noxious stimuli to the lung tissue cause p53 activation in the alveolar epithelial cells, which then facilitates EMT, cell senescence, apoptosis and fibrosis. In addition, noxious stimuli also lead to p53 inactivation in lung fibroblasts, which modulates fibrosis by inhibiting cell apoptosis.

Several studies have confirmed that p53 expression is significantly increased in AT2 cells and while inhibited in lung fibroblasts under fibrotic conditions [98]. Yao et al. generated an AT2-specific Sin3a conditional knockout mouse line to establish a novel conditional AT2 cell senescence model. Compared with control mice, the deposition of collagen, protein expression of FN, CTGF, and α-SMA are significantly increased in lungs with knockout mouse, indicating that AT2 cell senescence drives pulmonary fibrosis. In addition, p53 knockout in AT2 cells has been proven to abolish Sin3a knockout-induced cell senescence and pulmonary fibrosis, suggesting that blocking p53-induced cell senescence in AT2 cells may be a new approval for the treatment of progressive pulmonary fibrosis [99]. In addition, abundant plasminogen activator inhibitor 1 (PAI-1) is found in both AT2 cells and fibroblasts of aged mice, PAI-1 can drive bleomycin (BLM) and doxorubicin-induced AT2 cell senescence and pulmonary fibrosis through activating p53/p21/Rb pathway-mediated cell cycle repression [100]. Both p21 and miR-34a have been reported to be direct downstream target genes of p53. A recent study in vivo and in vitro has proved that in addition to repress self-renewal of AT2 cells by inducing cell cycle arrest, the activation of p53-p21 pathway can disrupt the p300–β-catenin interaction and prevent AT2–to–AT1 cell differentiation, all of which leading to pulmonary fibrosis [101]. AEC-specific activation of p53-miR-34a feedback has been shown to promote pulmonary fibrosis by accelerating AEC apoptosis, while fibroblast-specific activation of p53-miR-34a significantly inhibits pulmonary fibrosis through promoting fibroblast apoptosis and senescence [102, 103]. Neuronal PAS domain protein 2 (NPAS2) is a novel target gene of p53, Chen et al. revealed that p53 transcriptionally activates NPAS2 to promote EMT of AT2 cells and BLM-mediated pulmonary fibrosis [104]. Another study also found that activation of p53/RMRP/miR122 feedback loop can activate Notch-dependent EMT to aggravate silica-induce pulmonary fibrosis [105]. MDM4, an endogenous inhibitor of p53, is strikingly upregulated in the fibrotic lesions of human IPF. Qu et al. found that MDM4 knockout mice showed elevated acetylation and activation of p53 in lung myofibroblasts, accompanied by increased apoptotic myofibroblasts and reduced FN, α-SMA, collagen production. These results indicate that MDM4 exacerbates pulmonary fibrosis by inhibiting p53-dependent myofibroblast apoptosis [106]. Therefore, targeting p53 is a potential therapeutic strategy for pulmonary fibrosis (Fig. 4).

### Roles of p53 in cardiac fibrosis

Cardiac fibrosis is a common pathological final pathway in chronic myocardial disease and heart failure [107]. It is characterized by excessive deposition of ECM in the myocardium, hypertrophy and apoptosis of cardiomyocyte, distortion of myocardium architecture [108]. The activated fibroblasts and myofibroblasts are the primary effectors and source of matrix proteins during cardiac fibrosis [109]. Cardiomyocytes comprise 70-80% of the cellular volume in adult mammalian heart. The senescence of cardiomyocytes plays a crucial role in the development of cardiac fibrosis. The senescent cardiomyocytes exhibit impaired telomere shortening, increased pacing frequency, and reduced energy metabolism, all of which promote the release of SASP factors [110]. The released SASP factors, including TGF-β, tumour necrosis factor α, interleukin-6, can promote chronic inflammation, fibroblast and myofibroblast activation, and collagen deposition, thereby resulting in myocardial hypertrophy and progressive cardiac fibrosis [111, 112].

**Fig. 5:**
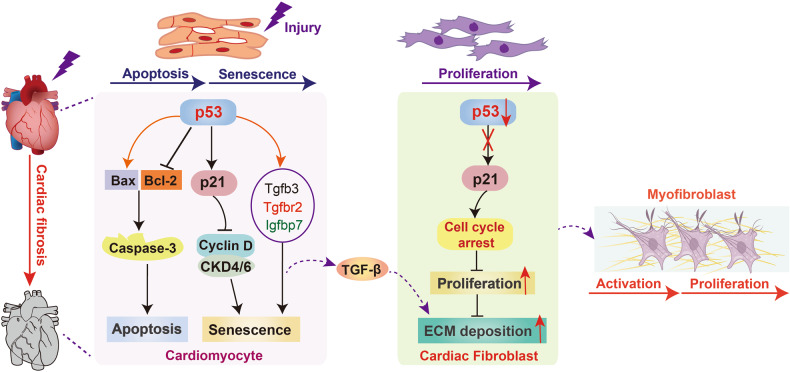
Roles of p53 in cardiac fibrosis. Activated p53 in cardiomyocytes evokes cell senescence and apoptosis, whereas inactivated p53 in cardiac fibroblasts causes cell proliferation, all of which promote the activation of myofibroblasts and development of cardiac fibrosis.

Recently, a large body of evidence has shown that p53 is involved in the pathogenesis of cardiac fibrosis. Pfithrin-α and fortilin are two effective inhibitors of p53, which can significantly alleviate diabetes mellitus and pressure overload-induced cardiac fibrosis by inhibiting the apoptosis and senescence of cardiomyocytes [113, 114]. Similarly, Nomura et al. constructed cardiomyocyte-specific p53 deletion mice and confirmed that p53 activation in cardiomyocytes is critical for the cardiomyocyte senescence and heart failure progression, accompanied by reduced TGF-β signaling-related molecules including TGF-β3, TGF-βR2, and insulin-like growth factor-binding protein-7 (IGFBP7) [115, 116]. These results suggest that p53-mediated cardiomyocyte apoptosis and senescence are important mediators in cardiac fibrosis. Notably, Liu et al. found that cardiac fibroblast-specific p53 conditional knockout mice with left ventricle pressure overload exhibited an exaggerated fibrotic response, including reduced cell cycle arrest, excessive fibroblasts accumulation, and ECM deposition, suggesting that p53-dependent fibroblast proliferation is involved in the pathogenesis of cardiac fibrosis [117]. Inhibition of nonspecific alkaline phosphatase (TNAP) suppresses cardiac fibroblast proliferation and myocardial infarction (MI)-induced cardiac fibrosis, which can be abrogated by pfithrin-α, indicating that TNAP inhibitor exerts an anti-fibrotic mechanism partially through p53 [118]. Moreover, MiR-125b can act as a novel inductor of cardiac fibrosis, which significantly promotes angiotensin II-induced cardiac fibroblast proliferation and cardiac fibrosis through inhibiting p53 activity [119]. There is increasing evidence that endothelial-to-mesenchymal transition (EndMT) plays a fundamental role in the pathogenesis and progression of cardiac fibrosis, while endothelial-specific deletion of p53 has been proved to inhibit cardiac fibrosis by suppressing EndMT and ECM production [120, 121]. Du et al. found that the plakoglobin-dependent cooperation of p53 with HIF-1α and Smad3 obviously activates TGF-β/Smad pathway and aggravates EndMT, thereby leading to cardiac fibrosis [122]. In conclusion, the above evidence suggests that p53 plays a significant role in the development of cardiac fibrosis (Fig. 5).

## Targeting p53 for organ fibrosis therapy

Fibrosis is a complicated process driven by numerous cell types and molecular processes. Numerous studies have demonstrated that p53 may serve as a potential therapeutic target for organ fibrosis. However, p53 is differentially regulated and has different functions in different cell types during fibrosis. Nanoparticle (NP) based drug delivery systems, cell targeting peptides (CTPs), and cell-penetrating peptides (CPPs) have shown to be effective targeting therapies for organ fibrosis. Here, we will discuss these updated cell-type specific targeting techniques and the role of cell-type specific targeting p53 in the treatment of organ fibrosis.

The common NP-based drug delivery systems include organic and inorganic NPs, and have numerous advantages such as targeting efficiency and minimal toxicity. Organic NPs are biodegradable and biocompatible, mainly include liposomes, polymeric micelles, and polylactic-co-glycolic acid (PLGA). The regular inorganic NPs are gold, mesoporous silica, silver, carbon nanotubes, and quantum dots. Bai et al. designed loading melatonin with PLGA and simultaneously extracted the activated HSC membranes to develop inflammation-responsive cell membrane-camouflaged nanoparticle, which has been demonstrated to effectively prevent the progression of hepatic fibrosis [123]. Targeting specific cells is one of the most significant problems in NP-based drug delivery to enhance delivery efficacy. Liposomal formulations that designed base on asialoglycoprotein receptor or mannose-6-phosphate receptor have been proved to target hepatocytes or HSCs in the liver tissue [124]. Further research found that cRGDyK-guided liposomes exhibited high selectivity toward activated HSCs. Li et al. constructed cRGDyK-modified sterically stable liposomes and formed activated HSC-specific drug delivery system, which can significantly inhibit the activation of HSCs and ultimately attenuate hepatic fibrosis [125]. Ji et al. developed a reduced glutathione-responsive NP-based drug delivery system to target activated cardiac fibroblasts, further in vivo study verified that the system improves the therapeutic efficacy of MI-induced cardiac fibrosis with lower systemic toxicity [126]. Another research demonstrated that 7 nm folic acid-conjugated gold nanoparticle can effectively inhibit renal fibrosis through targeting to renal tubule cells [127]. These results suggested that cell-type specific targeting therapy strategy may provide an effective treatment for organ fibrosis.

CTPs are short peptides comprising of 3–14 amino acids that have high affinity and specificity to cells or tissue targets. CTPs are recognized for their simple amino acid composition, easy synthesis, low immunogenicity, and toxicity [128]. Marudamuthu et al. developed a seven–amino acid deletion fragment of caveolin-1–derived peptide (CSP7), which has recently been used in fibrosis-related studies. In vitro testing with primary lung fibroblasts from lungs with end-stage IPF patients and IPF mice, CSP7 treatment exhibited reduced collagen I and α-SMA expression. In BLM-induced mice, CSP7 treatment can alleviate pulmonary fibrosis by targeting lung fibroblasts and AECs, respectively. On the one hand, CSP7 directly acts on AECs to inhibit AEC apoptosis via blocking p53. On the other hand, CSP7 can also act on fibroblasts to inhibit fibrotic lung fibroblast activation by restoring p53 [129].

CPPs are peptides that typically consisting 5–30 amino acids and can pass through tissue and cell membranes by energy-dependent or -independent mechanisms [130]. CPPs are classified into cell-specific and non-cell-specific peptides, which generally investigated to enhance or inhibit certain defined routes of intracellular signal transduction [131]. Baar et al. synthesized a cell-permeable FOXO4-p53–interfering peptide (FOXO4-DRI), which has been proved to selectively and effectively eliminate senescent cells by triggering p53 nuclear exclusion and aggravating cell-intrinsic apoptosis [132]. Senescence-like fibroblasts induced by radiation possess fibrotic phenotypes as myofibroblasts, Further study found that FOXO4-DRI can target senescence-like fibroblasts, increase non-small cell lung cancer cells’ radiosensitivity, inhibits the expression of α-SMA and collagen I, these results suggest that blocking p53 significantly reduces radiation-induced pulmonary fibrosis by targeting senescence-like fibroblasts [133].

In recent years, p53 has emerged as a promising therapeutic target and plays diverse roles in different cell types during organ fibrosis. As noted above, although cell-type specific targeting p53 have already been studied in pulmonary fibrosis, the specific targeting medicines of p53 and extensive clinical studies in other organ fibrosis are still lacking. The development of safe cell-type specific targeting medicine of p53 may be an effective therapy for organ fibrosis and a major goal for future research.

## Concluding remarks and future perspectives

The tumor suppressor p53 acts a vital role in both normal physiological function and diseases. Mounting evidence indicates that p53 participates in regulating organ fibrosis. As a result, there is significant interest in developing strategies that target p53 for the treatment of organ fibrosis. However, despite the abundant evidence of p53’s importance in organ fibrosis, many questions still remain unanswered. Firstly, the roles of p53 in organ fibrosis are cell type-specific, which means its functions can vary depending on the specific type of cell involved (Table 1). Secondly, the precise upstream regulators and downstream target genes of p53 during organ fibrosis have yet to be fully explored. The diversity and specificity of p53’s functions pose significant challenges in elucidating the regulatory mechanisms of p53 in organ fibrosis, as well as in developing relevant drug prevention and treatment programs. Consequently, further research should prioritize addressing these aforementioned problems to expedite the development of novel and promising therapeutic drugs that target p53 for the treatment of organ fibrosis.

**Table 1:** Summary of the p53 involved in organ fibrosis.

Disease	Model	Intervention	Biological effect	Refs.
Renal fibrosis	UUO-induced	p53 overexpression, p53 shRNA p53 siRNA, p53 inhibitor pfithrin-α	Activation of p53-p21 axis arrests cells at G2/M phase, prompts the partial EMT and inflammation of RTECs, thereby facilitating ECM production and renal fibrosis	[66, 134]
Renal fibrosis	IRI-induced, repeated low-dose cisplatin-induced	Proximal tubule cell-specific p53 knockout (PT-p53^-/-^)	Activation of p53 accelerates proximal tubule cell necrosis, apoptosis, and inflammation to promote renal fibrosis	[69, 70]
Renal fibrosis	Streptozotocin-induced diabetic nephropathy	Proximal tubule cell-specific p53 knockout (PT-p53^-/-^)	Activation of p53 binds to ZEB1-AS1, inhibits ZEB1-AS1 and ZEB1 expression to promote ECM accumulation and renal fibrosis	[71]
Renal fibrosis	Streptozotocin-induced diabetic nephropathy	Proximal tubule cell-specific p53 knockout (PT-p53^-/-^)	Activation of p53/microRNA-214 inhibits ULK1 and renal tubular autophagy, thereby promoting renal fibrosis	[72]
Hepatic fibrosis	Atherogenic-thioacetamide-induced	Hepatocyte-specific p53 knockout (H-53^-/-^), p53 siRNA	Activation of p53 in hepatocytes represses miR-17-92, induce CTGF synthesis to promote hepatic fibrosis	[85]
Hepatic fibrosis	CCl_4_-induced	p53 inhibitor pfithrin-α	Activation of SOCS1/p53 inhibits SLC7A11, accelerates ROS accumulation, promotes HSCs ferroptosis to inhibit hepatic fibrosis	[91]
Hepatic fibrosis	CCl_4_-induced	HSC-specific p53 knockout (HSC-53^-/-^)	Activation of p53 in HSCs promotes HSCs senescence, thereby ameliorating hepatic fibrosis	[92]
Hepatic fibrosis	CCl_4_-induced	p53 overexpression	Overexpression of p53 inhibits HSCs proliferation, suppresses cell cycle progression, promotes HSCs apoptosis to inhibit hepatic fibrosis	[135]
Hepatic fibrosis	Bile duct ligation-induced	p53 shRNA	Activation of p53-SLC25A28 axis aggravates mitochondrial iron accumulation, lipid peroxidation, and iron-dependent ferroptosis, thereby inhibiting hepatic fibrosis	[136]
Hepatic fibrosis	Fructose-induced	p53 siRNA	Activation of miR-34a/SIRT1/p53 axis promotes hepatocyte EMT to inhibit hepatic fibrosis	[137]
Pulmonary fibrosis	BLM-induced	p53 overexpression, p53 knockout (p53^-/-^)	Inhibition of p53 in lung fibroblasts expedites ECM production to promote pulmonary fibrosis	[98]
Pulmonary fibrosis	BLM-induced	p53 overexpression, p53 knockout (p53^-/-^)	Activation of p53 in AT2 cells accelerates cell apoptosis to promote pulmonary fibrosis	[98]
Pulmonary fibrosis	Sin3a knockout-induced	AT2-specific p53 knockout (AT2-p53^-/-^)	Activation of p53 in AT2 cells accelerates cell senescence to promote pulmonary fibrosis	[99]
Pulmonary fibrosis	Silica-induced	p53 siRNA, p53 knockout (p53^-/-^)	Activation of p53/RMRP/miR122 feedback loop accelerates EMT to promote pulmonary fibrosis	[105]
Pulmonary fibrosis	BLM-induced	p53 inhibitor MDM4, MDM4 siRNA, MDM4 overexpression	Inhibition of MDM4 in lung myofibroblasts activates p53 to promote myofibroblasts apoptosis and inhibit pulmonary fibrosis	[106]
Cardiac fibrosis	Pressure overload-induced	p53-cardiac fibroblast knockout (CF-p53^-/-^)	Deletion of p53 in fibroblasts exaggerates fibroblasts proliferation to facilitate cardiac fibrosis	[117]
Cardiac fibrosis	MI-induced	p53 inhibitor pfithrin-α	Activation of p53-p21 axis arrests cells cycle, suppresses proliferation of myofibroblasts and promotes cell senescence to inhibit cardiac fibrosis	[118]
Cardiac fibrosis	Pressure overload-induced	p53-cardiac endothelial knockout (CE-p53^-/-^)	Activation of p53 in endothelial cells triggers cell death and rarefaction of cardiac microvasculature to promote cardiac fibrosis	[121]
